# Genetic characteristics of *Bursaphelenchus xylophilu*s third-stage dispersal juveniles

**DOI:** 10.1038/s41598-021-82343-9

**Published:** 2021-02-16

**Authors:** Qiaoli Chen, Ruizhi Zhang, Danlei Li, Feng Wang

**Affiliations:** 1 Key Laboratory of Alien Forest Pests Detection and Control-Heilongjiang Province, School of Forestry, Northeast Forestry University, Harbin, 150040 Heilongjiang People’s Republic of China; 2 Key Laboratory of Sustainable Forest Ecosystem Management-Ministry of Education, Northeast Forestry University, Harbin, 150040 Heilongjiang People’s Republic of China

**Keywords:** Computational biology and bioinformatics, Genetics

## Abstract

The third-stage dispersal juvenile (DJ3) of pinewood nematode (PWN) is highly associated with low-temperature survival and spread of the nematode. Oil-Red-O staining showed that its lipid content was significantly higher compared with other PWN stages. Weighted gene coexpression network analysis identified that genes in the pink module were highly related to DJ3 induced in the laboratory (DJ3-lab). These genes were arranged according to their gene significance (GS) to DJ3-lab. Of the top 30 genes with the highest GS, seven were found to be highly homologous to the cysteine protease family cathepsin 1 (CATH1). The top 30 genes with the highest weight value to each of the seven genes in the pink module were selected, and finally 35 genes were obtained. Between these seven CATH1 homologous genes and their 35 highly related genes, 15 were related to fat metabolism or autophagy. These autophagy-related genes were also found to be highly correlated with other genes in the pink module, suggesting that autophagy might be involved in the mechanism of longevity in DJ3 and the formation of DJ3 by regulating genes related to fat metabolism.

## Introduction

The pinewood nematode (PWN), *Bursaphelenchus xylophilus*, is a migratory endoparasitic species that causes pine wilt disease (PWD), which is one of the most damaging diseases that affect conifer forests (particularly *Pinus* spp.)^1–3^. It has caused enormous ecological and financial losses by killing native pine species in Asia, North America, and Europe^2,4–11^. PWD was first discovered in the Purple Mountain of Nanjing in China in 1982. In a few short years, the occurrence of this disease was also reported in Jiangsu, Anhui, Shandong, Zhejiang, Guangdong, Hubei, Hunan, Taiwan, Hong Kong, etc^10^. According to the Announcement of the State Forestry and Grassland Administration (no. 4, 2019), this nematode has been found in the northeastern areas of China, where the lowest temperatures in winter are below − 20 °C, indicating that the disease is able to spread to low-temperature areas of China.

PWNs are transmitted to host trees by *Monochamus* beetles. PWNs have both phytophagous and mycophagous phases of development and a unique life cycle with four propagative and two dispersal juvenile stages^5,12,13^. When the environment is suitable, PWNs lifecycle is composed of eggs, four propagative juvenile stages and adults^13^. When conditions are adverse, the molting of the second-stage propagative juvenile (J2) formed a specific third-stage dispersal juvenile (DJ3)^14–16^. DJ3 can survive under unfavorable conditions, such as starvation, low temperature and dehydration until a certain level, inside the dead wood of host trees from autumn to the following spring^15^. When stimulated by the presence of the vector beetle, DJ3 molt to become the fourth-stage dispersal juvenile (DJ4 or dauer juveniles)^13,17^. DJ4 move and settle beneath the elytra or within the trachea of the beetles and are transported to another food source, thus spreading the disease to healthy pine trees^18,19^.

There have been a few reports on the factors associated with the formation and departure of DJ4^20–24^. However, compared with the DJ4 stage, the DJ3 stage has not been sufficiently investigated^16,25,26^. DJ3 is the prerequisite of DJ4, which can be transmitted with *Monochamus* beetles. Blocking the formation of DJ3 also blocks the formation of DJ4, thus preventing the nematodes from dispersing with *Monochamus* beetles and reducing the spread of PWD to healthy pine forests. Therefore, the elucidation of the mechanism of the formation of DJ3 is necessary to fully understand and control the epidemic of PWD.

The DJ3 stage is morphologically and functionally similar to the dauer stage of the model organism *Caenorhabditis elegans*, which is a long-lived stress-resistant stage^26,27^. Unlike DJ4, which have unique morphological features^28^, DJ3 are not morphologically distinct from third-stage propagative juveniles (J3), except that the body of DJ3 is slightly larger and accumulates many lipid droplets^13,16,26^. In previous studies, the percentage of DJ3 in standing trees in the field increased with time, reaching 60–100% by winter^15,29^, which might be related to their survival in a low-temperature environment, indicating a greater risk of its spread to low temperature areas. Therefore, additional studies are urgently needed to illustrate the mechanism of DJ3 formation and PWN survival under low temperatures. In this study, DJ3 induced in the laboratory (DJ3-lab) was compared with DJ3 collected in the field (DJ3-field) and other PWN stages, and the morphological characteristics of DJ3 were identified. Weighted gene coexpression network analysis (WGCNA) was used to explore the complex relationships between genes and phenotypes, which helped in determining the main functions of genes in the modules related to DJ3^30^.

## Results

### Dividing nematode stages by length and lipid content

**Figure 1:**
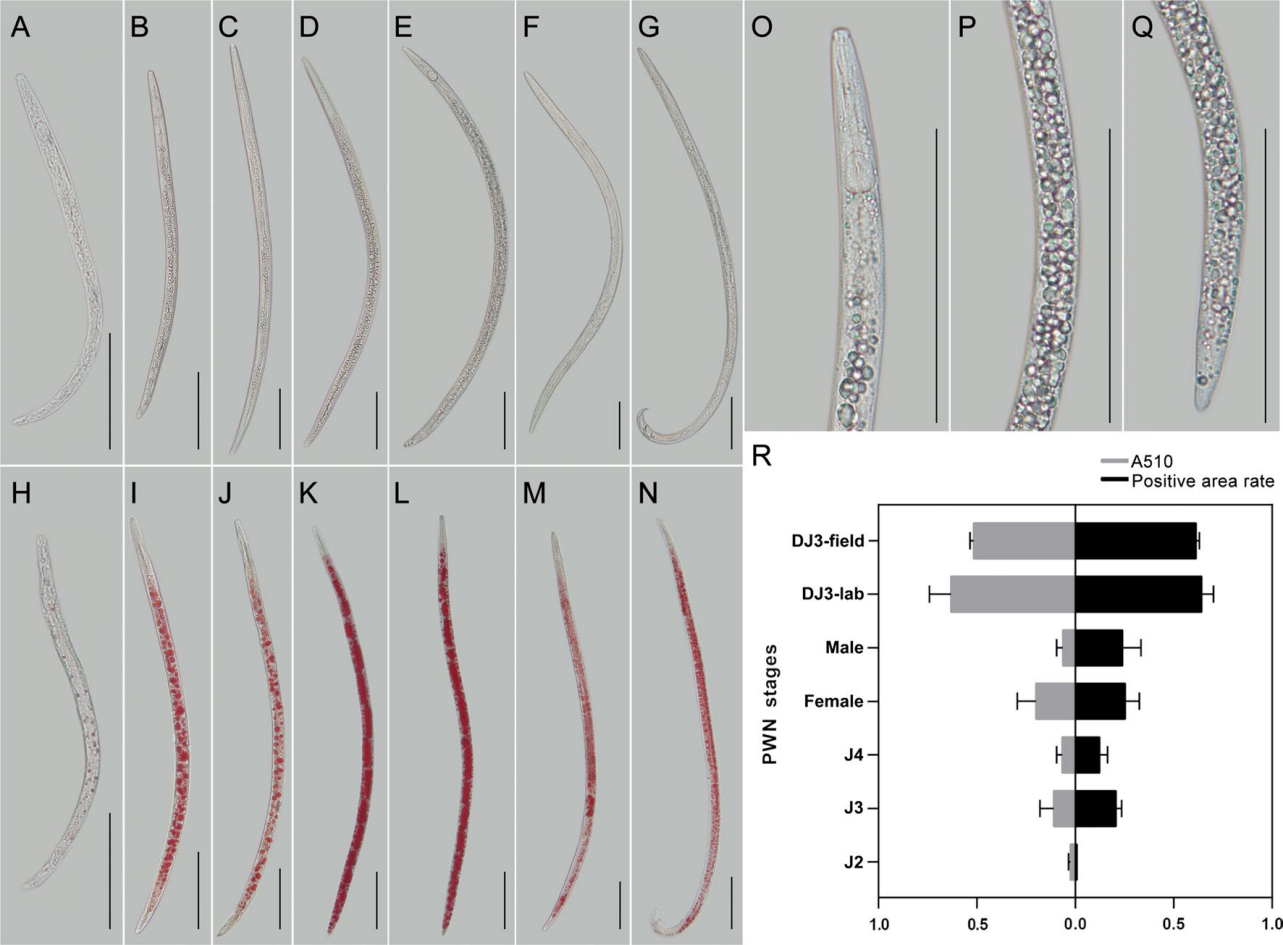
Morphological characteristics of different stages of PWNs. (**A**–**G**) Microscopy of J2, J3, J4, DJ3-lab, DJ3-field, Female, and Male. (**H**–**N**) Oil-Red-O stained J2, J3, L4, DJ3-lab, DJ3-field, Female, and Male. (**O**–**Q**) Details of DJ3-lab. (**R**) Comparison of the lipid content between different stages of PWNs, as determined by Oil-Red-O staining. Bars indicate 100 μm.

The morphometric values of J2; J3; fourth-stage propagative juvenile (J4); DJ3-lab; DJ3-field; female adult (Female); and male adult (Male) are summarized in Table 1. DJ3-lab and DJ3-field were similar to each other (Table 1, Fig. 1A–Q). Stored neutral lipids were assayed by the standard assay for adipocyte fat storage using Oil-Red-O staining (Fig. 1H–N). Student’s *t*-test was used to determine the significance between DJ3-lab and other stages. The results indicated that except for DJ3-field (*p-*value > 0.1), the lipid contents of other stages were significantly different from those of DJ3-lab (*p-*value < 0.0001). Oil-Red-O staining of the DJ3-lab and DJ3-field PWNs showed obviously more fat masses than other stages (Fig. 1O–Q), this characteristic can be used for the morphological identification of DJ3 (Fig. 1R).

**Table 1:** Morphometrics of different stages of PWNs. All measurements are in *μ*m and in the form: mean ± s.d. with (range).

Character	J2	J3	J4	DJ3-lab	DJ3-field	Female	Male
*n*	30	30	30	30	30	30	30
*L*	248 ± 32.25 (208–343)	486 ± 101.80 (292–688)	780 ± 66.18 (667–885)	816 ± 47.27 (719–875)	747 ± 32.96 (677–792)	1079 ± 72.44 (948–1292)	895 ± 86.19 (740–1052)
*a*	25 ± 0.53 (24–31)	29 ± 0.79 (25–37)	36 ± 1.44 (29–40)	43 ± 0.67 (37–50)	43 ± 0.47 (36–48)	39 ± 1.02 (33–47)	44 ± 1.23 (38–48)
*b*	5.2 ± 0.15 (4.4–6.4)	6.8 ± 0.22 (5.5–7.5)	8.5 ± 0.45 (7.3–10.8)	10.1 ± 1.09 (8.7–11.5)	10.0 ± 0.79 (8.6–11.7)	10.4 ± 1.35 (8.9–12.4)	8.7 ± 0.86 (7.9–11.9)
*c*	17 ± 0.78 (15–21)	20 ± 1.03 (16–27)	23 ± 1.55 (17–31)	21 ± 1.37 (18–25)	22 ± 1.44 (19–27)	26 ± 1.38 (22–32)	26 ± 1.11 (21–31)
*stylet*	12.5 ± 0.52 (11.4–13.6)	12.7 ± 1.10 (12.2–13.6)	13.6 ± 0.46 (12.2–14.4)	12.7 ± 1.10 (11.2–13.6)	12.6 ± 0.82 (11.4–13.5)	14.4 ± 0.78 (14.2–15.4)	14.4 ± 0.55 (14.2–15.4)

### Digital gene expression (DGE) sequencing

To characterize the gene transcript patterns, 27 libraries (three replicates for each stage: J2, J3, J4, Female, Male, the second-stage propagative juvenile prior to DJ3-lab (J2-2), DJ3-lab, DJ3-field, and DJ4 of PWNs were constructed and sequenced. After removing the low-quality reads, an average of 23.42 Mb clean reads were obtained for each sample (Table S1). The dataset was deposited in the Sequence Read Archive (SRA; accession: SRR9990585; BioProject ID: PRJNA560635). A total of 17,811 assembled unigenes were generated from the 27 libraries.

### Weighted gene coexpression analysis (WGCNA)

**Figure 2:**
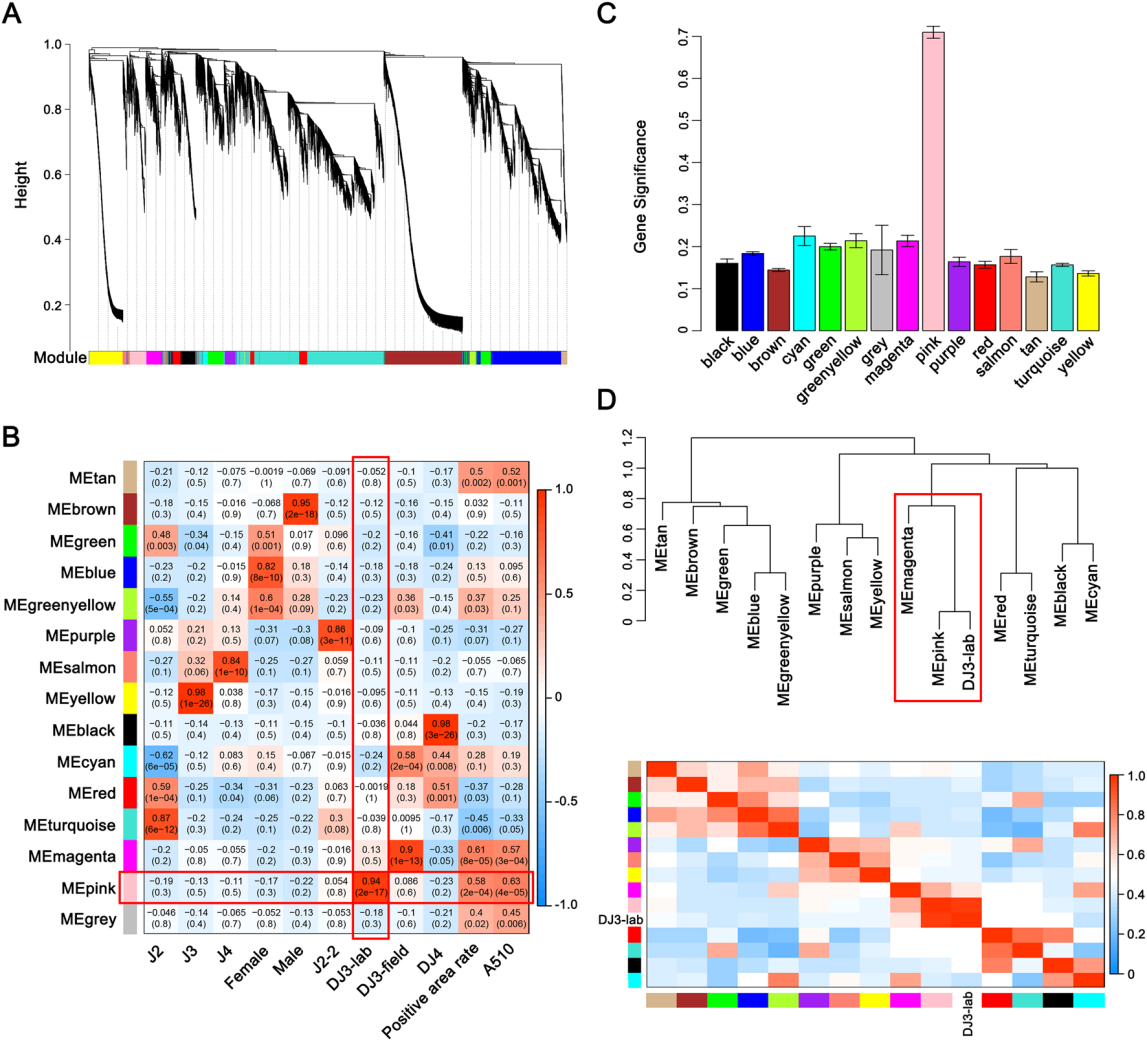
Weighted gene coexpression analysis (WGCNA) revealed the module highly related to *Bursaphlenchus xylophilus* DJ3-lab. (**A**) Clustering dendrograms of genes, with dissimilarity based on the topological overlap, together with assigned module colors. (**B**) Module-trait associations. Each row corresponds to an ME, and each column corresponds to a trait. Each cell contains the corresponding correlation and *p-*value. The table is color-coded by correlation according to the color legend. (**C**) Bar plot of module significance defined as the mean gene significance across all genes in the module to DJ3-lab. (**D**) The hierarchical clustering dendrogram and the heatmap of correlated MEs and DJ3-lab.

To eliminate noise from genes that were not expressed, we filtered probes with median FPKM levels that did not exceed 1.0 and obtained 15,863 genes. The expression values of these 15,863 genes in 27 transcriptomes of PWNs were used to construct the coexpression module with WGCNA package tools. Cluster analysis was performed on these samples to determine whether there were any obvious outlier (Fig. S1). We choose power 6, which was the lowest power at which the scale-free topology fit index curve flattened out upon reaching a high value (in this case, approximately 0.85, Fig. S2), to construct coexpression modules. Fourteen distinct gene coexpression modules were identified (Fig. 2A). These coexpression modules were constructed and shown in different colors, and the number of genes in each module was shown in Table S2. The gene network was visualized by plotting a heatmap (Fig. S3). Each row and column of the heatmap corresponds to a single gene. The heatmap depicts adjacencies or topological overlaps, with light colors denoting low adjacency (overlap) and dark colors denoting high adjacency (overlap).

The module eigengene (ME) is defined as the first principal component of a given module. It can be considered a representative of the gene expression profiles in a module (Fig. S4). Modules with common expression pattern interactions in the coexpression modules that were associated with particular traits (Fig. S1) were identified based on the correlation between the ME and trait (Fig. 2B). The analysis identified two modules (pink and magenta) associated with lipid content. Of these two modules, the pink module was significantly associated with DJ3-lab (correlation value, cor = 0.94, *p-*value = 2 × 10^–17^, Fig. 2B,C), and the magenta module was significantly associated with DJ3-field (cor = 0.90, *p-*value = 1 × 10^–13^, Fig. 2B and Fig. S5).

We concentrated on DJ3-lab as the trait of interest. To study the relationships among the identified modules, the MEs were used as representative profiles, and ME correlation was used to quantify module similarity. The trait that determined whether the PWNs were DJ3-lab was added to the MEs to see how this trait fit into the ME network. The dendrogram indicated that the pink module had the highest association with DJ3-lab and that the pink and magenta modules were highly related (Fig. 2D).

### Functional and pathway enrichment analyses of genes in interesting modules

Gene Ontology (GO) enrichment and Kyoto Encyclopedia of Genes and Genomes (KEGG) enrichment were performed on the genes in the pink and magenta modules. There was no significant difference in the GO terms and pathways in which the two modules were enriched (Fig. S6-S9). Genes in these two modules were mainly enriched in GO: 0044699 (single-organism process), GO: 0008152 (metabolic process), GO: 0003824 (catalytic activity), GO: 0005488 (binding), GO: 0005623 (cell), and GO: 0044464 (cell part). They were mainly enriched in global and overview maps (ko01100 and ko01200), lipid metabolism (ko00590, ko00561 and ko00071), transport and catabolism (ko04142, ko04146 and ko04140), carbohydrate metabolism (ko00040, ko00010, ko00053 and ko00620), and xenobiotics biodegradation and metabolism pathways (ko00982, ko00980 and ko00983). The above results indicated that although there were differences in gene expression between DJ3-field and DJ3-lab as DJ3-field was induced by complex environmental conditions, the genes highly related to the two have similar functions and played a role in similar pathways.

### Module visualization and candidate genes selection

**Figure 3:**
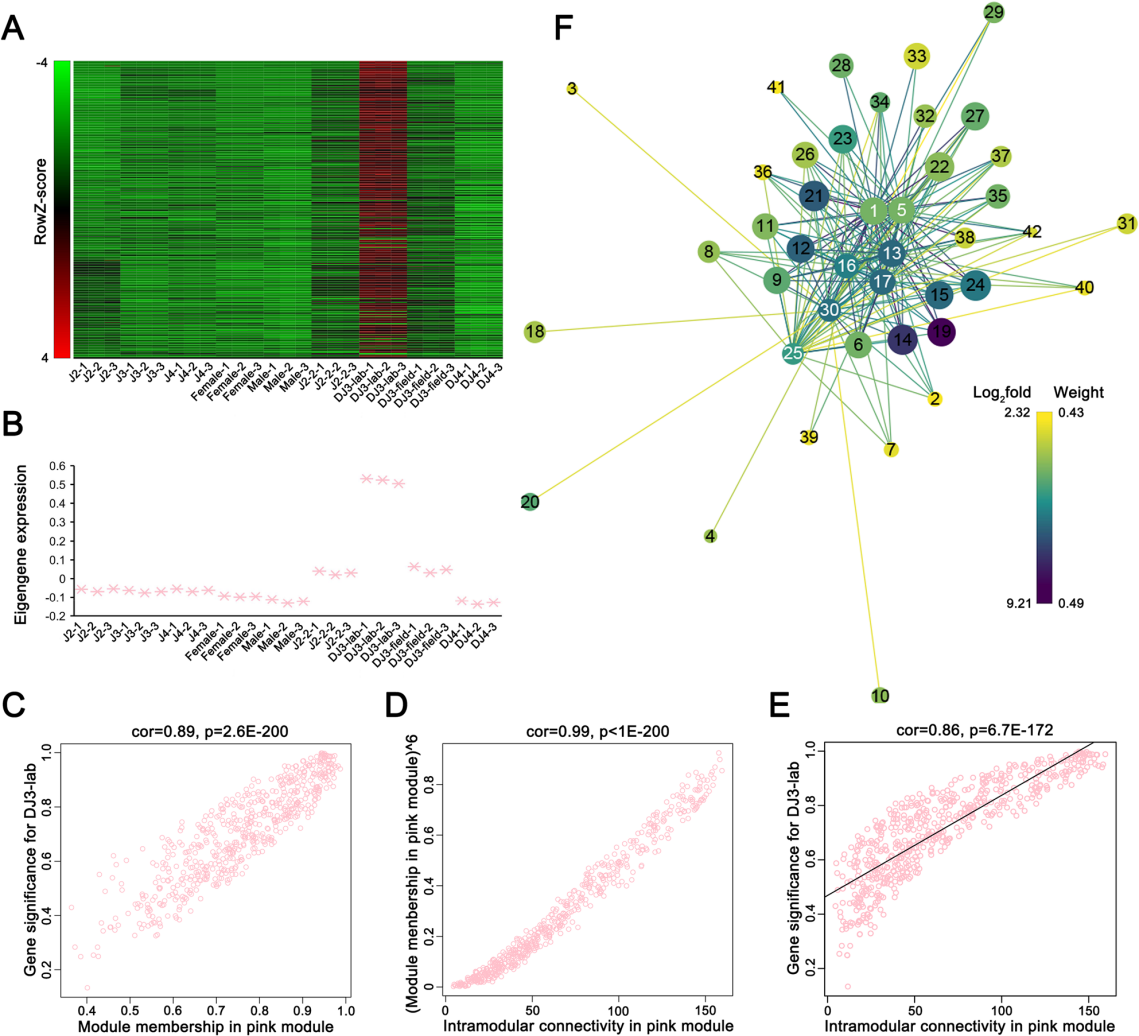
Weighted gene coexpression analysis (WGCNA) revealed a gene-network module enriched in *Bursaphlenchus xylophilus* DJ3-lab. (**A**) Heatmap of the pink module genes (rows) across the samples (columns). (**B**) The pink module eigengene expression values (y-axis) across the samples (x-axis) (**C**) GS for DJ3-lab vs. MM in the pink module. (**D**) MM raised to power 6 (y-axis) vs. IC (x-axis) for the pink module. (**E**) GS (y-axis) vs. IC (x-axis) for the pink module. (**F**) Gene network for the 7 selected genes in the pink module with the top 30 highest GS to DJ3-lab (detailed in Table S4 and Table S5). Prefuse Force Directed Layout was applied based on the weight value between two genes. The size of the dots represents GS (from 0.9361 to 0.9984). The color of the dots represents log_2_fold (from 2.3238 to 9.2053) for the FPKM of DJ3-lab vs. the average FPKM of the other samples. The colors of the lines represent the weight value between two genes (from 0.4330 to 0.4921). The label of dots is listed based on IC (from 97.0445 to 159.5326), and the 7 selected dots are highlighted with white color labels.

We concentrated on the genetic characteristics of DJ3-lab as this stage was induced in the laboratory under certain conditions. An expanded view of the expression of all genes in the pink module was compared with the ME expression of the pink module across all samples (Fig. 3A,B). The ME took on low values in arrays where many module genes were underexpressed (green color in the heatmap). The ME took on high values in arrays where many module genes were overexpressed (red in the heatmap). The results indicated that in addition to the high expression in DJ3-lab, the genes in the pink module also had higher expression in samples J2-2 and DJ3-field than in other samples (Fig. 3A,B). This suggested that the genes in the pink module could also be highly correlated with the formation of DJ3.

To quantify the similarity of genes in the pink module, the associations of individual genes with the DJ3-lab trait were quantified by defining gene significance (GS) as the correlation between the gene and the trait, and a quantitative measure of module membership (MM) was also defined as the correlation of the ME and the gene expression profile for the module. A scatterplot of GS vs. MM in the pink module was plotted, and GS and MM were highly correlated, illustrating that genes highly significantly associated with a trait were often also the most important (central) elements of modules associated with a trait (Fig. 3C).

The intramodular connectivity (IC) value was defined only for the genes inside a given module. IC measures how connected, or co-expressed, a given gene is with respect to the genes of a particular module. IC may be interpreted as a measure of MM. The IC for each gene in the pink module was calculated. Genes with the top 30 highest IC values were considered intramodular hub genes in this study (Table S3, Fig. S10). The amino acid sequences of 20 of these genes could be aligned to highly homologous sequences in the nr library. Six of them had high homology with the cysteine protease family cathepsin 1 (CATH1) from *B. mucronatus* (Table 2, GenBank: AID50178.1). These 6 hub genes in the pink module are represented in red in Fig. S10. After raising the MM to a power of 6, it was highly correlated with the IC (Fig. 3D). Highly connected intramodular hub genes tend to have high MM values to the respective module. The GS vs. IC was also plotted, and we observed that genes with high IC tended to have high GS (Fig. 3E).

**Table 2:** Genes in the pink module that have high homology to CATH1 and the top 30 highest IC or GS values.

Gene annotation	Blast nr *p-*value	IC	Hub gene	GS	GS *p-*value	MM	MM *p-*value
*cath1-6*	3.45 × 10^–108^	159.532607	Yes	0.98884616	1.05 × 10^–29^	0.97416231	1.49 × 10^–23^
*cath1-4*	1.11 × 10^–90^	149.365747	Yes	0.98799769	3.62 × 10^–29^	0.95314787	3.14 × 10^–19^
*cath1-5*	1.31 × 10^–107^	154.86967	Yes	0.98652294	2.57 × 10^–28^	0.96839503	4.38 × 10^–22^
*cath1-3*	1.05 × 10^–41^	147.251794	Yes	0.98346067	8.14 × 10^–27^	0.95032175	8.33 × 10^–19^
*cath1-1*	6.95 × 10^–76^	147.475827	Yes	0.98060788	1.19 × 10^–25^	0.95107981	6.45 × 10^–19^
*cath1-7*	5.13 × 10^–15^	140.082666	No	0.974976	8.71 × 10^–24^	0.941333	1.31 × 10^–17^
*cath1-2*	5.51 × 10^–82^	144.119954	No	0.97129291	8.74 × 10^–23^	0.94612349	3.20 × 10^–18^
*cath1-8*	3.33 × 10^–103^	145.147244	Yes	0.9319333	1.53 × 10^–16^	0.9593262	2.98 × 10^–20^

Of the top 30 genes with the highest GS to the DJ3-lab in the pink module, the amino acid sequences of 17 of them could be aligned to homologous sequences in the nr library. Seven of them had very high homology with CATH1 from *B. mucronatus* (Table 2). These seven genes basically overlapped with the six hub genes mentioned above, except that two of them (*cath1-7* and *cath1-2*) were not hub genes, and one hub gene (*cath1-8*) was not among them. The top 30 genes with the highest weight value to each of the 7 genes in the pink module were selected, and finally 35 genes were obtained. The network data were exported to Cytoscape (Fig. 3F) by Prefuse Force Directed Layout based on the weight value between two genes. The whole network contained 210 regulatory relationships of 42 genes (detailed in Table S4 and Table S5).

**Figure 4:**
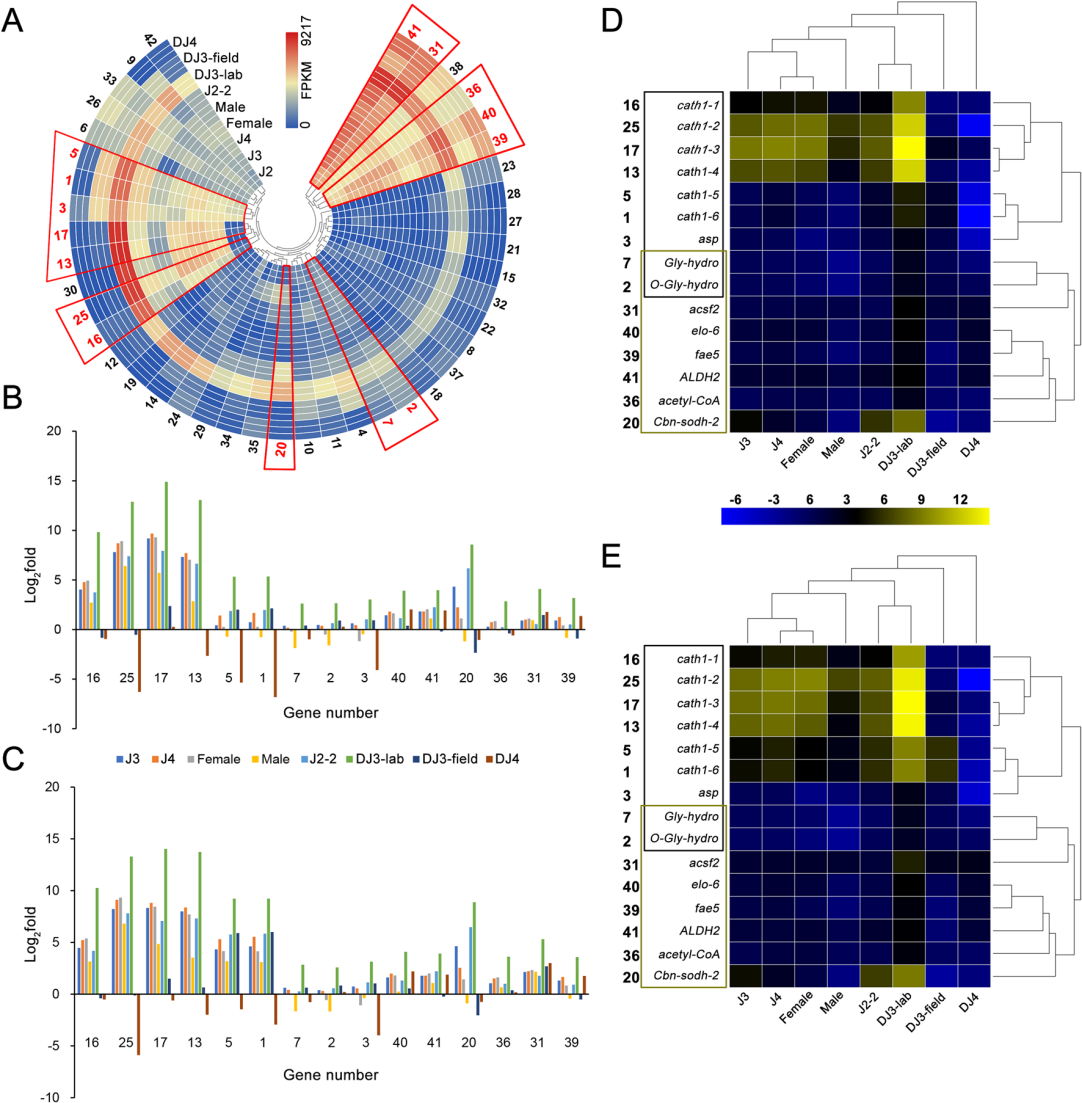
The expression pattern of 7 CATH1 homologous genes with the top 30 highest GS values to the DJ3-lab and their top 30 related genes in the pink module. (**A**) The expression (FPKM) of 42 genes in the pink module include 7 CATH1 homologous genes with the top 30 highest GS values to the DJ3-lab and their top 30 related genes. Genes are clustered by their expression pattern. The 15 genes associated with lipid metabolism or autophagy are framed in red. (**B**) Comparison of the relative transcript abundances of the 15 lipid metabolism- or autophagy-associated genes from different PWN stages (normalized by J2) by FPKM. (**C**) Comparison of the relative transcript abundances of the 15 lipid metabolism- or autophagy-associated genes from different PWN stages (normalized by J2) by RT-PCR. (**D**) Heatmap and clustering analysis of the results from (**B**). (**E**) Heatmap and clustering analysis of the results from (**C**). Genes in the black box are associated with autophagy, while those in the khaki box are associated with lipid metabolism.

These 42 genes were numbered by IC and selected for further study (Fig. 4A, Table S4). Among them, 8 (*elo-6*, *ALDH2*, *Cbn-sodh-2*, *acetyl-CoA*, *acsf2*, *fae5*, *O-Gly-hydro30*, *cath1-4 and Gly-hydro*) were related to different lipid metabolism-related pathways (ko00062, ko01212, ko00061, ko00071, ko01040, ko00561, ko00590 and ko00600). Nine of them (*cath1-1*, *cath1-2*, *cath1-3*, *cath1-4*, *cath1-5*, *cath1-6*, *Gly-hydro*, *O-Gly-hydro30* and *asp*) were related to autophagy-related pathways (ko04140, ko04142, ko04210 and ko04974). In total, there were 15 potential candidate genes (Fig. 4A, highlighted in red), including 6 of the 7 selected genes in the pink module with high GS to DJ3-lab and aligned to the CATH1 (*cath1-5*, *cath1-6*, *cath1-1*, *cath1-2*, *cath1-3* and *cath1-4*).

### Candidate gene RT-PCR and their relationships with each other

The 15 genes mentioned above that were associated with the lipid metabolism-related pathway or autophagy-related pathway were selected for further study. Their expression levels between different stages of PWNs were further verified by RT-PCR (Fig. 4B,C). Gene expression levels of these genes from J2 were used for data normalization. Student’s *t*-test was used to detect significant differences between the two stages. The results indicated that the expression levels of genes from DJ3-lab were most significantly different from those from DJ3-field and DJ4 (*p-*value < 0.0001); were significantly different from those from Female and Male (0.0001 < *p-*value < 0.01); and were different from those from J3, J4 and J2-2 (0.01 < *p-*value < 0.05). The expression levels of genes from J3, J4, Female, Male and J2-2 were not obviously different (*p-*value > 0.05) from each other, but they were all different from those from DJ3-field and DJ4 (*p-*value < 0.05), except for genes from Male, which were not obviously different from those from DJ3-field (*p-*value > 0.5). The expression levels of genes from DJ3-field were different from those from DJ4 (0.01 < *p-*value < 0.05).

The clustering analysis of the 15 genes and different PWN stages indicated that J2-2 and DJ3-lab had a similar expression pattern, while DJ3-field and DJ4 were quite different from other stages (Fig. 4D,E). Additionally, the expression patterns of genes with similar functions were relatively uniform. Moreover, we noticed that although the expression levels of *cath1-2*, *cath1-3* and *cath1-4* were relatively high in the other PWN stages, they had an unmatched high expression level in the DJ3-lab stage (*p-*value < 0.0001, Fig. 4D,E). However, the expression levels of these 3 genes were relatively low in DJ3-field and DJ4. These results indicated that these 3 genes might have a more important role in laboratory-cultured nematodes.

**Figure 5:**
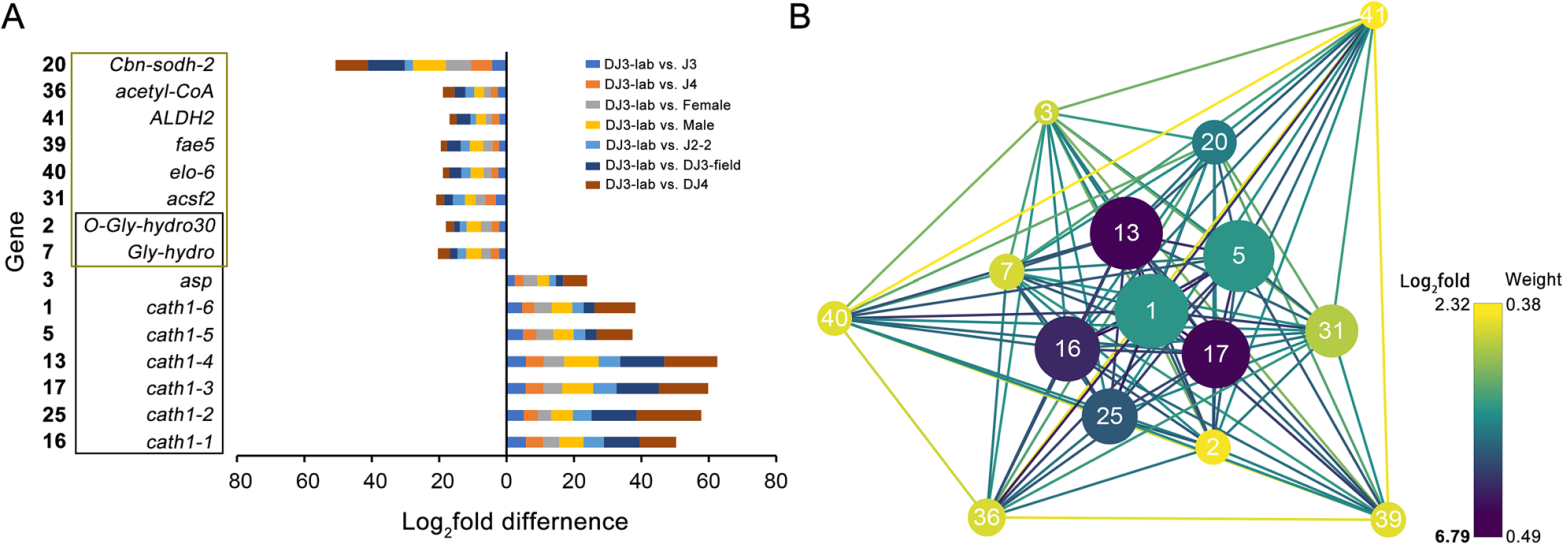
Gene expression difference and network for the 15 lipid metabolism- or autophagy-associated genes selected from the pink module. (**A**) The FPKM differences (normalized by J2) between DJ3-lab and the other PWN stages. Genes in the black box are associated with autophagy, while those in the khaki box are associated with lipid metabolism. (**B**) Gene network for the 15 candidate genes. The size of the dots represents GS (from 0.9361 to 0.9984). Prefuse Force Directed Layout was applied based on the weight value between two genes. The color of the dots represents log_2_fold (from 2.3238 to 6.7868) for the FPKM of DJ3-lab vs. the average FPKM of the other samples. The color of the lines represents the weight value between the two genes (from 0.3770 to 0.4864). The label of dots is listed based on IC (from 108.1303 to 159.5326).

For the 15 selected genes, the differences between DJ3-lab and the other PWN stages in the expression of genes related to autophagy were relatively larger than those for genes related to lipid metabolism (*p-*value < 0.0001, Fig. 5A). For each of the 15 genes, its weight value with the other 14 genes was selected, and their network data were exported to Cytoscape by Prefuse Force Directed Layout based on the weight values between two genes. The whole network contained 105 regulatory relationships of 15 genes (Fig. 5B, details can be found in Table S4 and Table S5). The results indicated that the genes related to autophagy might be related to more genes in the pink module, as they had relatively higher IC values and weight values with other genes. Those genes also had large differences in gene expression between DJ3-lab and the other PWN stages, and they had larger GS values to DJ3-lab than those of the genes related to lipid metabolism. These results illustrated that these autophagy-related genes might play an important role in DJ3-lab and they were closely related to lipid metabolism-related genes.

## Discussion

Recently, PWNs have been found in northeastern areas of China, where the lowest temperatures in winter are below − 20 °C, indicating that the large areas of pine forest in the north might be at risk of infection. Therefore, it is necessary to study the ability of this nematode to resist to diverse climatic conditions. The DJ3 stage, which is also a long-lived stress-resistant stage, might play an important role in the process of spreading northward, as DJ3s can survive under unfavorable conditions, such as starvation, low temperature and dehydration, inside the dead wood of host trees from autumn to the following spring^4,15^. Additionally, DJ3 is the prerequisite of DJ4, which can be transmitted with *Monochamus* beetles. Therefore, blocking DJ3 should prevent the spread of the nematode.

Unlike DJ4, which have unique morphological features, DJ3 are not morphologically different from J3 (Table 1, Fig. 1A–Q), except that the body of DJ3 is slightly larger and accumulates many lipid droplets^13,16^. In this study, Oil-Red-O staining was performed to highlight the differences in the lipid content among all PWN stages. DJ3-lab were compared to DJ3-field and the other PWN stages cultured in the lab. The results indicated that DJ3-lab and DJ3-field had obviously more fat masses than other stages. Because sometimes the difference in body length is not obvious between PWNs, the lipid content may be a better choice for distinguishing DJ3 from other stages.

WGCNA was then used to analyze the genetic characteristics of DJ3-lab and explore the complex relationships between gene profiles and phenotypes. The results showed that the genes highly related to DJ3-lab (pink module) and DJ3-field (magenta module) had similar functions and played a role in similar pathways. In addition, the expression of genes highly expressed in DJ3-lab (pink module) was also higher in J2-2 and DJ3-field than in other stages of PWNs (Fig. 3A,B). These results indicated that although there were differences in gene expression between DJ3-field and DJ3-lab since DJ3-field was induced by complex environmental conditions, DJ3 might be characterized by a high expression of genes with the same type of function, and those genes might increase in expression before DJ3 is formed. Therefore, these genes may be highly related to DJ3 and may be target genes for preventing the formation of DJ3.

We then evaluated these genes in the pink module and screened these genes based on IC and GS because IC represents the connection strength within the module, and GS represents the correlation between expression profiles and traits. In the top 30 genes based on IC or GS, there were multiple genes with high homology to the CATH1. We selected 7 genes with high homology to the CATH1 from the top 30 highest GS value genes and then screened the top 30 highest weight value genes for each of these 7 genes in the pink module. In total, 42 genes with 210 regulations were obtained. Of these 42 genes, 15 were related to autophagy or lipid metabolism. These included 6 of the 7 genes that were highly homologous to the CATH1, and these 6 genes were all highly related to autophagy. Although the expression patterns of the functionally similar genes of the 15 genes were highly similar, there was a high correlation between the 15 genes. There was an increase in hub genes involved in autophagy-related genes, and these genes had a relatively higher correlation with other genes, suggesting that autophagy might play an important role in DJ3 to promote the formation of DJ3 and maintain its longevity by regulating genes related to lipid metabolism.

Under normal conditions, autophagy occurs at the basal level. However, it is accelerated by a variety of stresses, such as starvation^31^, the accumulation of abnormal proteins and organelle damage^32^. Thus far, accumulating evidence shows that the activation of autophagy seems essential for longevity^31,33–35^. Autophagic activity is commonly elevated in many long-lived animals and is essential for their longevity, suggesting that autophagy is one of the convergent downstream mechanisms of all these longevity paradigms^32^. It has been reported that lipid turnover by autophagy is essential for the longevity and clearance of lipids (lipophagy) and mitochondria (mitophagy) and are related to aging in *C. elegans*^36–38^. Therefore, autophagy might also be the longevity mechanism of DJ3. However, the process by which autophagy in PWNs is activated and is involved with lipid metabolism to induce the nematode to accumulate lipids and prolong life requires further investigation.

## Materials and methods

### Nematodes

The experimental PWNs were collected from Dalian, Liaoning Province, China, on June 9, 2017. The PWNs were cultured on *Botrytis cinerea* and placed in a 25 °C incubator without light for different periods to obtain different stages of propagative juveniles. Artificially induced DJ3-lab were cultured on *B. cinerea* according to the method of Ishibashi and Kondo with a few modifications^14^. Field materials of DJ3-field and DJ4 were collected from Dalian, Liaoning Province, China, on April 10, 2017 and June 9, 2017, respectively. The Baermann funnel method was used to extract nematodes in different stages. For each stage of PWN, 30 nematodes were photographed using a microscope Bx51 (Olympus, Japan). The lengths of the nematodes in the pictures were measured by flexible ropes and converted to real lengths^39^. Three separate biological replicates were performed.

### Determination of the nematode neutral lipid content

Lipids in PWNs are stored mainly as triglycerides and other neutral lipids. Oil-Red-O staining^40^ was performed using the Improved Oil-Red-O Stain Kit (Leagene, China, catalog number DL0011) as described in the manual. Dyed PWNs in different stages (J2; J3; J4; Female; Male; DJ3-lab; DJ3-field and DJ4) were all divided into two parts, which were used for photographing and lipid content determination.

Images of dyed PWNs were captured with a microscope Bx51 (Olympus, Japan). The quantification of Oil-Red-O staining was performed by image analysis. ImageJ was used to separate each color image into its RGB channel components. The green channel was used for further analysis^41,42^, and the total dyed areas of the PWNs were calculated. A minimum of 9 PWNs were measured for each stage, and 3 separate biological replicates were performed. Significance was determined by Student’s *t*-test.

The quantification of the lipid content was also assessed by extracting the dye from stained PWNs, and 1 ml of isopropyl alcohol was added to each centrifuge tube with stained nematodes. The extracted dye was removed and then measured with a GeneQuant 1300 ultraviolet spectrophotometer (Biochrom Ltd., UK), and its absorbance was monitored spectrophotometrically at 510 nm^41^. A total of 1000 PWNs were measured for each stage each time, and three separate biological replicates were performed. Significance was determined by Student’s *t*-test.

### DGE sequencing

Total RNA was extracted separately from the powders of different stages of PWNs using TRIzol (Invitrogen, USA, cat. no. 15596-026)^43^. The RNA Nano 6000 Assay Kit for the Agilent 2100 Bioanalyzer system (Agilent, USA) was used to examine the concentration, RNA integrity number (RIN), 28S/18S and fragment size of the total RNA. The purity of RNA was determined by a NanoDrop spectrophotometer (Thermo Scientific, USA). Genomic DNA was removed using DNase I, Amplification Grade (Invitrogen, USA, cat. no. 18068-015).

Each RNA sample was sheared and reverse transcribed using random primers to obtain cDNA for library construction. The construction of the libraries and sequencing were all performed on a BGISEQ-500 RNA-seq platform (BGI, Shenzhen, China), and 50-bp single-end (SE) reads were generated. SOAPnuke (v1.5.2, https://github.com/BGI-flexlab/SOAPnuke) was used to filter the generated raw sequencing reads. Clean reads were mapped to the reference genome of the PWN (BioSample: SAMEA2272519), which is available at NCBI (http://www.ncbi.nlm.nih.gov/assembly/310678), using HISAT2 (v2.0.4, http://www.ccb.jhu.edu/software/hisat)^44^. Clean reads were then mapped to reference sequences using Bowtie2 (v2.2.5, http://bowtie-bio.sourceforge.net/Bowtie/index.shtml)^45^.. The matched reads were calculated and normalized to fragments per kilobase per million mapped fragments (FPKM) using RSEM (v1.2.12, http://deweylab.biostat.wisc.edu/RSEM)^46^.

### WGCNA

WGCNA was used to explore the complex relationships between genes and phenotypes^30^. An extensive overview of WGCNA, including numerous tutorials, can be found at http://www.genetics.ucla.edu/labs/horvath/CoexpressionNetwork/. In this study, we aimed to construct coexpression modules using the expression data of genes from different stages of PWNs. The lipid content of PWNs and the stage to which they belonged were selected as traits. The appropriate power value was determined when the degree of independence was over 0.8. The minimum number of genes was set as 30 for highly reliable results. WGCNA was implemented in the R software package (http://www.r-project.org/), and the heatmap tool package was implemented to analyze the strength of the interactions. Module-trait associations were estimated using the correlation between the ME and the trait. The IC was calculated for each gene by summing the connection strengths with other module genes and dividing this number by the maximum IC. For each expression profile, GS was calculated as the absolute value of the Pearson correlation between the expression profile and each trait. MM was defined as the Pearson correlation of the expression profile and each ME. Network depictions of interesting modules were constructed with Cytoscape software^47^.

### GO and KEGG enrichment analyses of selected modules

GO^48^ and KEGG^49^ enrichment analyses were performed on genes in selected modules. The results of the analysis were extracted, and a *p-*value ≤ 0.05 after the correction was used as the threshold. The top 10 records were extracted if there were more than 10 records.

### RT-qPCR

The transcript levels of differentially expressed genes for different stages of PWNs were measured by RT-qPCR using the GoTaq 2-Step RT-qPCR System Kit (Promega, USA, cat. no. A6010) and the Stratagene Mx3000P qPCR system (Agilent, USA). The primers used in this study are listed in Table S6. The RT-qPCR results were normalized as log_2_(JX/J2)-fold changes with a constitutively expressed gene, *mec-12*, as an internal control, which has no significant difference in expression level at each stage. The normalization of data was performed according to the instructions of the GoTaq 2-Step RT-qPCR System Kit, and the ${2}^{-\mathrm{\Delta \Delta }{C}_{T}}$ method was used to analyze the data^50^. Three separate biological replicates were performed, and each replicate was assessed three times. Significance was determined by Student’s *t*-test.

## Supplementary Information

Supplementary Information
