# Supplementary material for: Genetic characteristics of Bursaphelenchus xylophilus third-stage dispersal juveniles
Source: Sci Rep. 2021 Feb 16;11:3908. doi: 10.1038/s41598-021-82343-9 (PMC7887269; doi:10.1038/s41598-021-82343-9)
Supplement: Supplementary file 1 — Supplementary Information [file 41598_2021_82343_MOESM1_ESM.pdf]

Genetic characteristics of *Bursaphelenchus xylophilus* third-stage dispersal juveniles

Qiaoli Chen[1,2§], Ruizhi Zhang[1§], Danlei Li[1,2], Feng Wang[1,2*]

[1]Key Laboratory of Alien Forest Pests Detection and Control-Heilongjiang Province, School of Forestry, Northeast Forestry University, Harbin 150040, Heilongjiang, P. R. China; [2]Key Laboratory of Sustainable Forest Ecosystem Management-Ministry of Education, Northeast Forestry University, Harbin 150040, Heilongjiang, P. R. China;

[*]Corresponding author.

[§]These authors contributed equally to this work.

E-mail addresses: qiaolichen@nefu.edu.cn (Q. Chen), zhangruizhi@nefu.edu.cn (R. Zhang), danleili@nefu.edu.cn (D. Li), fengwang@nefu.edu.cn (F. Wang).

Table S1 Statistical analysis of the RNA sequencing data.

Table S2 Number of genes in each module.

Table S3 Genes with the top 30 highest IC values in the pink module.

Table S4 Forty-Two network genes for the pink module.

Table S5 Top 30 genes with the highest weight value to each of the 7 genes.

Table S6 Primers used in this study.

Fig. S1 Clustering dendrogram of samples based on their Euclidean distance.

Fig. S2 Analysis of network topology with various soft-thresholding powers.

Fig. S3 Visualization of the gene network using a heatmap plot.

Fig. S4 Module eigengene expression profile.

Fig. S5 Bar plot of module significance defined as the mean gene significance across all genes in the module to the DJ3-field trait.

Figure S6. GO enrichment analysis (Biology Process) of the genes in the pink and magenta modules.

Figure S7. GO enrichment analysis (Cellular Component) of the genes in the pink and magenta modules.

Figure S8. GO enrichment analysis (Molecular Function) of the genes in the pink and magenta modules.

Figure S9. KEGG enrichment analysis of the genes in the pink and magenta modules.

Figure S10. Visualization of the pink module (weight>0.3).

Table S1 Statistical analysis of the RNA sequencing data.

Samples	Clean Reads Number	Clean Reads Rate (%)	Clean Reads Q20 (%)	Gene Mapping Rate (%)
J2-1	22,133,728	99.62	97.50	71.27
J2-2	22,203,673	99.94	97.38	71.64
J2-3	24,112,070	99.90	97.94	71.58
J3-1	24,057,741	99.67	97.20	75.77
J3-2	24,145,341	99.56	97.64	74.66
J3-3	23,985,362	99.48	97.67	75.13
J4-1	24,037,515	99.59	96.90	76.96
J4-2	23,465,363	99.65	97.64	76.68
J4-3	24,013,521	99.56	97.68	76.84
Female-1	23,960,379	99.26	95.30	78.06
Female-2	24,013,135	99.46	97.34	78.99
Female-3	23,961,244	99.75	97.42	78.56
Male-1	21,359,122	99.41	94.80	76.86
Male-2	22,063,153	99.53	96.57	76.96
Male-3	21,624,233	99.75	97.31	76.66
J2-2-1	24,110,169	99.89	97.90	59.92
J2-2-2	23,951,367	99.39	97.86	64.57
J2-2-3	24,061,351	99.86	97.34	62.44
DJ3-lab-1	24,018,211	99.51	97.10	75.94
DJ3-lab-2	23,996,384	99.42	97.00	74.01
DJ3-lab-3	23,940,211	99.80	98.40	73.51
DJ3-field-1	23,821,746	99.21	98.60	73.39
DJ3-field-2	23,971,254	99.12	98.41	73.89
DJ3-field-3	23,814,547	99.39	98.76	74.06
DJ4-1	22,051,038	91.38	98.44	66.51
DJ4-2	23,023,245	95.89	98.76	67.88

DJ4-3	22,415,547	96.76	98.42	67.69

Table S2 Number of genes in each module.

Module colors	Gene number
black	603
blue	2639
brown	2589
cyan	143
green	1010
greenyellow	234
grey	20
magenta	533
pink	583
purple	437
red	789
salmon	158
tan	202
turquoise	4775
yellow	1148

Table S3 Genes with the top 30 highest IC values in the pink module.

No.	Gene annotation	IC	Homologous protein GenBank ID	Protein name	Blast nr p-value
1	*cath1-6*	159.5326073	AID50178.1	cysteine protease family cathepsin 1 [*Bursaphelenchus mucronatus*]	3.4518×10^{-108}
2	*O-Gly-hydro30*	158.3084016	EPB74230.1	O-Glycosyl hydrolase family 30 [*Ancylostoma ceylanicum*]	1.59787×10^{-80}
3	*asp*	157.6912185	KJH53579.1	eukaryotic aspartyl protease [*Dictyocaulus viviparus*]	2.08533×10^{-23}
4	*gst-33*	155.5006374	ABN64198.1	glutathione S-transferase-1 [*Meloidogyne incognita*]	3.11943×10^{-22}
5	*cath1-5*	154.8696696	AID50178.1	cysteine protease family cathepsin 1 [*Bursaphelenchus mucronatus*]	1.31168×10^{-107}
6	*CBN-DHS-2*	154.3596573	EGT37950.1	CBN-DHS-2 protein [*Caenorhabditis brenneri*]	4.56842×10^{-68}
7	*Gly-hydro*	153.8170115	CDJ96960.1	*Glycoside hydrolase domain* containing protein [*Haemonchus contortus*]	4.85292×10^{-21}
8	*str-124*	153.2131533	CEF64053.1	7TM GPCR, serpentine receptor class r (Str) family-containing protein [*Strongyloides ratti*]	2.54895×10^{-06}
9	*gst-36*	152.0856442	KKA74463.1	Glutathione S-transferase [*Pristionchus pacificus*]	1.8039×10^{-15}
10	*irld-39*	151.0613212	-	-	-
11	*BUX.s01254.113*	150.5725348	-	-	-
12	*BUX.s01149.55*	150.4648858	-	-	-
13	*cath1-4*	149.3657473	AID50178.1	cysteine protease family cathepsin 1 [*Bursaphelenchus mucronatus*]	1.10675×10^{-90}
14	*BUX.s00460.305*	149.1136527	-	-	-
15	*scl-13*	148.7282799	ADG86239.1	venom allergen-like protein VAP3 [*Bursaphelenchus xylophilus*]	1.09812×10^{-120}
16	*BUX.s01149.58*	148.0852976	-	-	-
17	*cra-1*	147.8092805	XP_003147865.1	hypothetical protein LOAG_12304 [*Loa loa*]	7.65088×10^{-45}
18	*cath1-1*	147.4758269	AID50178.1	cysteine protease family cathepsin 1	6.95176×10^{-76}

				[*Bursaphelenchus mucronatus*]	
19	*cath1-3*	147.2517936	AID50178.1	cysteine protease family cathepsin 1 [*Bursaphelenchus mucronatus*]	1.05473×10^{-41}
20	*BUX.s00609.55*	146.8648011	EYB86268.1	hypothetical protein Y032_0282g1277 [*Ancylostoma ceylanicum*]	2.65302×10^{-149}
21	*BUX.s01281.196*	146.791013	-	-	-
22	*spp-20*	146.6361924	-	-	-
23	*BUX.s01149.57*	146.4990787	-	-	-
24	*Cbn-sodh-2*	145.7646357	EGT60304.1	CBN-SODH-2 protein [*Caenorhabditis brenneri*]	4.08829×10^{-91}
25	*BUX.s01254.340*	145.7577156	XP_003113617.1	hypothetical protein CRE_26128 [*Caenorhabditis remanei*]	2.91415×10^{-54}
26	*cath1-8*	145.1472441	AID50178.1	cysteine protease family cathepsin 1 [*Bursaphelenchus mucronatus*]	3.33012×10^{-103}
27	*BUX.s00773.12*	145.1195146	-	-	-
28	*Src-3*	144.8323753	CDJ86525.1	Src homology-3 and Tyrosine protein kinase domain containing protein [*Haemonchus contortus*]	2.25183×10^{-61}
29	*BUX.s01066.169*	144.7103838	-	-	-
30	*acs-14*	144.5409195	CEF60576.1	Acyl-CoA synthetase family member 2,mitochondrial [*Strongyloides ratti*]	3.21114×10^{-76}

Table S4 Forty-two network genes for the pink module.

No.	Gene annotation	IC	GS	MM	log_2fold	Homologous protein GenBank ID	Protein name	Blast nr p-value
1	*cath1-6*	159.5326073	0.988846164	0.974162311	4.446578165	AID50178.1	cysteine protease family cathepsin 1 [*Bursaphelenchus mucronatus*]	3.4518×10^{-108}
2	*O-Gly-hydro30*	158.3084016	0.950980428	0.98237167	2.390274998	EPB74230.1	O-Glycosyl hydrolase family 30 [*Ancylostoma ceylanicum*]	1.59787×10^{-80}
3	*asp*	157.6912185	0.94038912	0.987078893	2.78486744	KJH53579.1	eukaryotic aspartyl protease [*Dictyocaulus viviparus*]	2.08533×10^{-23}
4	*gst-33*	155.5006374	0.946208904	0.976789213	3.844799544	ABN64198.1	glutathione S-transferase-1 [*Meloidogyne incognita*]	3.11943×10^{-22}
5	*cath1-5*	154.8696696	0.986522944	0.968395033	4.445621127	AID50178.1	cysteine protease family cathepsin 1 [*Bursaphelenchus mucronatus*]	1.3117×10^{-107}
6	*CBN-DHS-2*	154.3596573	0.987017029	0.962918769	4.492759739	EGT37950.1	CBN-DHS-2 protein [*Caenorhabditis brenneri*]	4.56842×10^{-68}
7	*Gly-hydro*	153.8170115	0.951258729	0.973186585	2.697644098	CDJ96960.1	Glycoside hydrolase domain containing protein [*Haemonchus contortus*]	4.85292×10^{-21}
8	*str-124*	153.2131533	0.97307036	0.971150209	3.943376809	CEF64053.1	7TM GPCR, serpentine receptor class r (Str) family-containing protein [*Strongyloides ratti*]	2.54895×10^{-06}
9	*gst-36*	152.0856442	0.9882188	0.9581010	4.837761218	KKA74463.1	Glutathione S-transferase [*Pristionchus pacificus*]	1.8039×10^{-15}

			59	89				
10	*irld-39*	151.0613212	0.958553926	0.966832536	4.055685227	-	-	-
11	*BUX.s01254.113*	150.5725348	0.985323567	0.961525805	4.23168163	-	-	-
12	*BUX.s01149.55*	150.4648858	0.990383667	0.955363866	6.916516288	-	-	-
13	*cath1-4*	149.3657473	0.987997692	0.953147869	6.786765412	AID50178.1	cysteine protease family cathepsin 1 [*Bursaphelenchus mucronatus*]	1.10675×10^{-90}
14	*scl-13*	148.7282799	0.996755148	0.950599458	7.988188299	ADG86239.1	venom allergen-like protein VAP3 [*Bursaphelenchus xylophilus*]	1.0981×10^{-120}
15	*BUX.s01149.58*	148.0852976	0.991397361	0.951453104	6.715878015	-	-	-
16	*cath1-1*	147.4758269	0.980607884	0.951079809	6.198012395	AID50178.1	cysteine protease family cathepsin 1 [*Bursaphelenchus mucronatus*]	6.95176×10^{-76}
17	*cath1-3*	147.2517936	0.98346067	0.950321745	6.730701785	AID50178.1	cysteine protease family cathepsin 1 [*Bursaphelenchus mucronatus*]	1.05473×10^{-41}
18	*BUX.s01281.196*	146.791013	0.972281584	0.959713911	3.669566309	-	-	-
19	*BUX.s01149.57*	146.499	0.991	0.948	9.20532	-	-	-

		0787	652369	356876	3331			
20	*Cbn-sodh-2*	145.7646357	0.960124154	0.958609479	4.896200997	EGT60304.1	CBN-SODH-2 protein [*Caenorhabditis brenneri*]	4.08829×10^{-91}
21	*BUX.s00773.12*	145.1195146	0.998200834	0.94505033	7.079933101	-	-	-
22	*Src-3*	144.8323753	0.994629495	0.947766482	4.105008558	CDJ86525.1	Src homology-3 and Tyrosine protein kinase domain containing protein [*Haemonchus contortus*]	2.25183×10^{-61}
23	*BUX.s01066.169*	144.7103838	0.992362662	0.944095388	5.38064778	-	-	-
24	*BUX.s00713.823*	144.3383328	0.998358989	0.943168968	6.398065219	-	-	-
25	*cath1-2*	144.1199535	0.971292913	0.946123493	5.448167269	AID50178.1	cysteine protease family cathepsin 1 [*Bursaphelenchus mucronatus*]	5.50549×10^{-82}
26	*Pank-1*	143.6341537	0.985966158	0.942157774	3.716878439	KHN77364.1	Pantothenate kinase 1 [*Toxocara canis*]	7.8244×10^{-146}
27	*asp-14*	142.7739429	0.991201239	0.945415366	4.67688499	-	-	-
28	*BUX.s01038.42*	142.5620508	0.979755113	0.951448053	4.54819314	-	-	-

29	*oac-29*	142.3434777	0.969728086	0.949773571	4.370195752	-	-	-
30	*cath1-7*	140.0826661	0.974976	0.941332997	6.579303266	AID50178.1	cysteine protease family cathepsin 1 [*Bursaphelenchus mucronatus]*	5.13471×10^{-15}
31	*acsf2*	140.0134726	0.968402614	0.934459428	3.048284161	KHN84146.1	Acyl-CoA synthetase family member 2, mitochondrial [*Toxocara canis*]	1.5861×10^{-180}
32	*BUX.s00110.118*	137.8320848	0.976563287	0.940996049	3.89475684	-	-	-
33	*sbp-1*	136.8748978	0.985616522	0.932652606	3.018632387	KHN86442.1	Sterol regulatory element-binding protein 1 [*Toxocara canis*]	2.28728×10^{-25}
34	*epg-5*	135.3618996	0.967264158	0.939019132	4.760012785	-	-	-
35	*BUX.s00773.15*	127.8206086	0.979798501	0.918791247	4.49593605	-	-	-
36	*acetyl-CoA*	124.5177749	0.953498858	0.916112818	2.6715104	CEF71468.1	Carboxyl transferase domain and Biotin [*Strongyloides ratti*]	0
37	*Ddram-1*	122.8930373	0.969978549	0.901733566	3.542053909	KHN72428.1	DNA damage-regulated autophagy modulator protein 1 [*Toxocara canis*]	7.98356×10^{-22}
38	*acetyl-coenzyme*	120.9009149	0.9690489	0.9004126	3.119458652	ERG80270.1	acetyl-coenzyme a cytoplasmic [*Ascaris suum*]	4.10816×10^{-53}

			17	35				
39	*fae5*	113.140987	0.951062062	0.884964575	2.581306214	EJW88645.1	fatty acid elongation protein 5 [*Wuchereria bancrofti*]	1.41793×10^{-84}
40	*elo-6*	112.9332421	0.950435931	0.883631275	2.58735458	XP_002633891.1	*C. briggsae* CBR-ELO-6 protein [*Caenorhabditis briggsae*]	4.38478×10^{-78}
41	*ALDH2*	108.1303498	0.94344528	0.876521463	2.323796869	KHN86680.1	Aldehyde dehydrogenase, mitochondrial [*Toxocara canis*]	0
42	*tmem-135*	97.04447902	0.936145496	0.861986689	2.896698411	NP_508800.2	TMEM (human TransMEMbrane protein) homolog [*Caenorhabditis elegans*]	1.24721×10^{-40}

Table S5 Top 30 genes with the highest weight value to each of the 7 genes.

No.	Number of genes	Number of genes	Weight value
1	21	1	0.492137315
2	14	1	0.48994234
3	19	1	0.489799172
4	1	24	0.488884436
5	15	1	0.487896545
6	13	1	0.486417876
7	13	1	0.486417876
8	12	1	0.486246042
9	1	38	0.486233078
10	1	22	0.485689578
11	30	1	0.485672596
12	30	1	0.485672596
13	17	1	0.485275634
14	17	1	0.485275634
15	16	1	0.482392852
16	16	1	0.482392852
17	1	35	0.48210381
18	9	1	0.481253274
19	1	27	0.480864513
20	13	6	0.480119286
21	1	26	0.480107618
22	1	11	0.479618217
23	17	6	0.479410971
24	30	6	0.479314339
25	32	1	0.479200002
26	23	1	0.478527701
27	6	1	0.478405224

28	21	5	0.477791704
29	25	1	0.477422573
30	25	1	0.477422573
31	34	1	0.476765546
32	16	6	0.476657717
33	14	5	0.476154436
34	1	5	0.475697258
35	1	5	0.475697258
36	5	24	0.475196268
37	19	5	0.474910561
38	15	5	0.474557232
39	1	36	0.474272405
40	13	12	0.473994688
41	30	12	0.473905776
42	30	9	0.473658176
43	13	9	0.47357636
44	17	12	0.473520511
45	37	1	0.473350111
46	13	38	0.473160544
47	17	9	0.473133572
48	13	19	0.473009983
49	13	21	0.472701187
50	5	22	0.472496483
51	13	5	0.472444688
52	13	5	0.472444688
53	25	6	0.472428799
54	13	14	0.472399877
55	12	5	0.47222922
56	28	1	0.472021812

57	13	15	0.471611443
58	13	24	0.471138116
59	30	13	0.47112965
60	30	13	0.47112965
61	16	9	0.470966846
62	16	12	0.470922726
63	5	38	0.470806323
64	17	13	0.470653266
65	17	13	0.470653266
66	17	5	0.470506191
67	17	5	0.470506191
68	30	14	0.470475606
69	1	33	0.470208337
70	17	14	0.470091778
71	30	5	0.469789444
72	30	5	0.469789444
73	1	42	0.469625388
74	29	1	0.469077662
75	5	35	0.468628749
76	13	16	0.468608779
77	13	16	0.468608779
78	16	5	0.468531731
79	16	5	0.468531731
80	5	27	0.467683275
81	16	14	0.467508077
82	25	9	0.467296145
83	9	5	0.467250861
84	25	12	0.467183058
85	5	26	0.467022205

86	17	38	0.466919423
87	1	41	0.466721319
88	5	11	0.4665363
89	17	19	0.466387739
90	30	15	0.466211256
91	17	15	0.466056341
92	23	5	0.465923046
93	17	30	0.465491621
94	17	30	0.465491621
95	17	21	0.465326736
96	30	19	0.465324422
97	25	13	0.465300432
98	25	13	0.465300432
99	6	5	0.465054954
100	13	11	0.464746157
101	16	38	0.464583938
102	13	22	0.464408186
103	15	16	0.464076411
104	32	5	0.464003618
105	16	19	0.463967446
106	17	24	0.463675797
107	17	11	0.463336156
108	30	16	0.463303646
109	30	16	0.463303646
110	25	5	0.463233787
111	25	5	0.463233787
112	13	26	0.463064092
113	30	11	0.463038036
114	17	16	0.462781084

115	17	16	0.462781084
116	25	14	0.462730003
117	13	35	0.462584436
118	21	16	0.462499118
119	37	5	0.462364065
120	34	5	0.461380517
121	16	11	0.46129075
122	16	24	0.461242755
123	5	36	0.460912942
124	13	36	0.460811926
125	13	37	0.46063131
126	30	21	0.460506513
127	25	15	0.459976306
128	17	25	0.459520409
129	17	25	0.459520409
130	13	2	0.459238335
131	25	16	0.458803845
132	25	16	0.458803845
133	30	24	0.458773489
134	13	34	0.458591772
135	13	23	0.458472272
136	17	2	0.458317814
137	13	27	0.458166764
138	30	2	0.458037937
139	5	33	0.457827981
140	28	5	0.457505494
141	25	19	0.457300119
142	5	42	0.457013271
143	13	8	0.456921826

144	25	11	0.456916109
145	13	42	0.456592284
146	13	32	0.456564496
147	29	5	0.45623694
148	17	22	0.456161126
149	16	2	0.455921579
150	17	8	0.455750052
151	17	26	0.455598375
152	13	40	0.455461228
153	30	8	0.455365744
154	5	41	0.455096719
155	13	39	0.45476396
156	17	35	0.454539959
157	16	36	0.454405726
158	16	22	0.453921104
159	16	26	0.453742428
160	17	23	0.453574309
161	17	7	0.453353921
162	17	36	0.453341156
163	30	23	0.45332145
164	30	7	0.453297609
165	16	8	0.452917617
166	30	26	0.452673928
167	17	37	0.452647503
168	17	34	0.45262933
169	25	38	0.452097024
170	30	25	0.451991066
171	30	25	0.451991066
172	16	7	0.451978798

173	16	35	0.451837711
174	25	2	0.45144753
175	25	21	0.451259305
176	16	37	0.451053463
177	30	22	0.45079755
178	23	16	0.450655766
179	25	7	0.450123176
180	25	24	0.450117136
181	17	27	0.450091837
182	17	32	0.449604669
183	16	34	0.449432102
184	16	42	0.449311923
185	17	42	0.448864586
186	25	8	0.447698177
187	17	40	0.447431316
188	16	40	0.447424374
189	16	27	0.447302278
190	30	38	0.446420502
191	16	39	0.446401037
192	25	26	0.445035837
193	25	23	0.444466987
194	30	27	0.444012965
195	25	36	0.443689919
196	30	4	0.442498894
197	25	22	0.442301326
198	30	31	0.441184503
199	30	18	0.439744592
200	25	42	0.438817696
201	30	3	0.437924211

202	25	35	0.437828917
203	25	34	0.437688685
204	25	37	0.437076831
205	30	20	0.436962621
206	25	31	0.436040908
207	30	10	0.435822749
208	25	40	0.4353366
209	25	27	0.434892645
210	30	29	0.433026623

Table S6 Primers used in this study.

Gene	Primer
cath1-1	F-`GGTCTTTCGCACAATCGCTC`
	R-`TTCAAACCACTCATCGCCGT`
cath1-2	F-`GCTGTGTCATCCGCTCATCA`
	R-`TTGGAGGCAAACTTCAGCGA`
cath1-3	F-`CCAAAACTTCCGCGAATCCA`
	R-`AGTGGTTAGCAGGTTTGGCT`
cath1-4	F-`GCTGTGTCATCCGCTCATCA`
	R-`TTGGAGGCAAACTTCAGCGA`
cath1-5	F-`ACATACCTTGATGCCGGACC`
	R-`ACCCCAGTCTTCTCCCCATT`
cath1-6	F-`CTCACCAGGAGTGTTGCGAT`
	R-`CATCGGGTGGGAGTTGTGTT`
Gly-hydro	F-`CAACGCTGAAAAACGGGCAA`
	R-`TCCGTAAAGGCTGCACCAAA`
O-Gly-hydro30	F-`TAACCCCCGGATCTCATCGT`
	R-`AGCCAGCCGGATCTTTGATT`
asp	F-`CCTGCGATGTGGACTGCTTA`
	R-`GCGGCTGATGTAAGTGTTCG`
elo-6	F-`GCTGTTTTCAGTATCGGCGG`
	R-`GCGGAGAACCAGGAAAATGG`
ALDH2	F-`AATATCGTGGCCCCTGAACC`
	R-`GCATCCTTTGCAGCCTTGAC`
Cbn-sodh-2	F-`TGGTTTTGGTGTCATTGCCG`
	R-`TCGTCCAGCGTCTCATTCAG`
acetyl-CoA	F-`TCGCCAAAAAGGTGGGCTAT`
	R-`ATGGCGGGGATGTTGATGTT`
acsf2	F-`GGAAAGTGGGAGTGTCAGCA`

	R-`GATGTTTGAGGTCGGGGAGG`
fae5	F-`AGTCGAGACCGAACCCTTTC`
	R-`TCTGATCCAAGTGGGGGTTG`
mec-12	F-`TTGAGCCCTACAACTCCGTG`
	R-`AAGCGGTAATGGAGCTGACC`

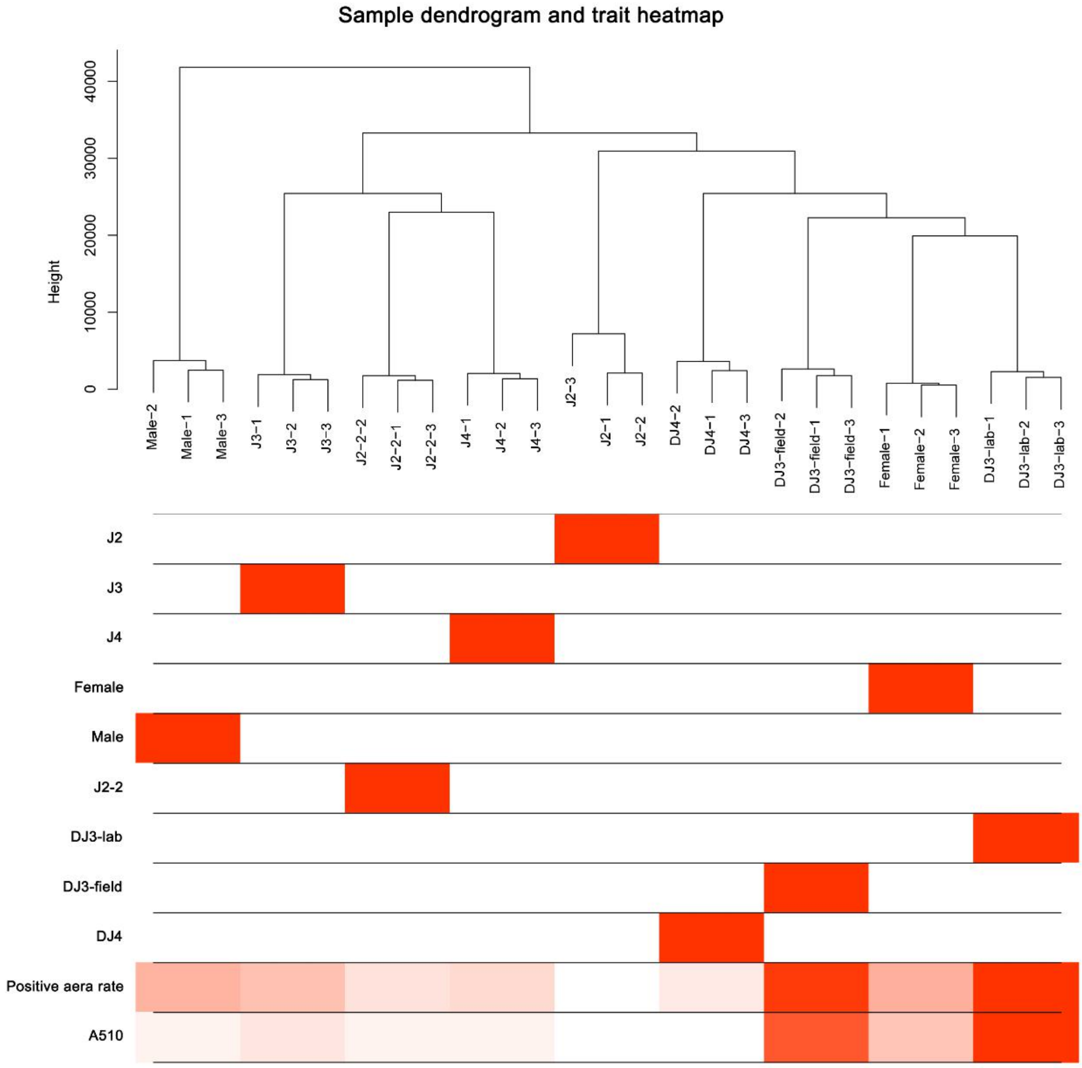

Figure S1. Clustering dendrogram of samples based on their Euclidean distance

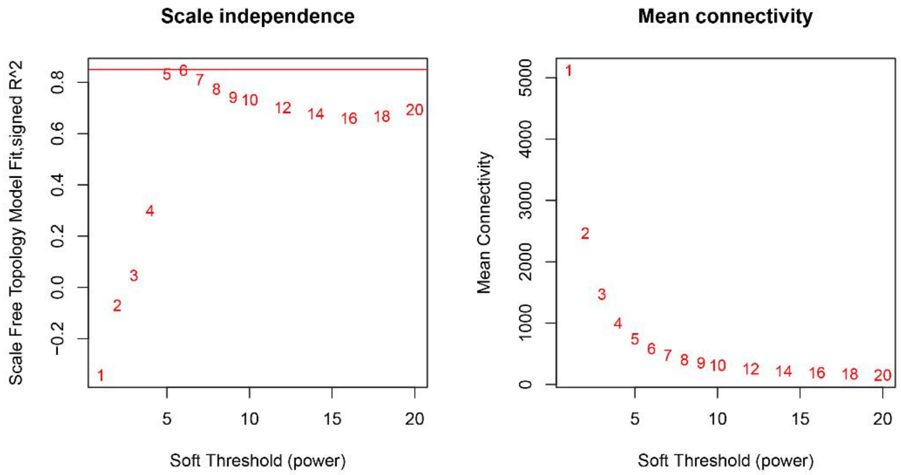

Figure S2. Analysis of network topology with various soft-thresholding powers. The left panel shows the scale-free fit index (y-axis) as a function of the soft-thresholding power (x-axis). The right panel displays the mean connectivity (degree, y-axis) as a function of the soft-thresholding power (x-axis). The red line indicates scale-free fit index is equal to 8.5.

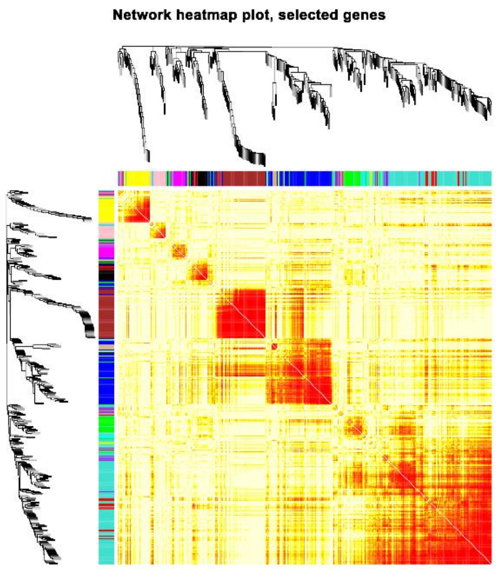

Figure S3. Visualization of the gene network using a heatmap plot. The heatmap depicts the Topological Overlap Matrix (TOM) among 400 genes in the analysis. Light color represents low overlap and progressively darker red color represents higher overlap. Blocks of darker colors along the diagonal are the modules. The gene dendrogram and module assignment are also shown along the left side and the top.

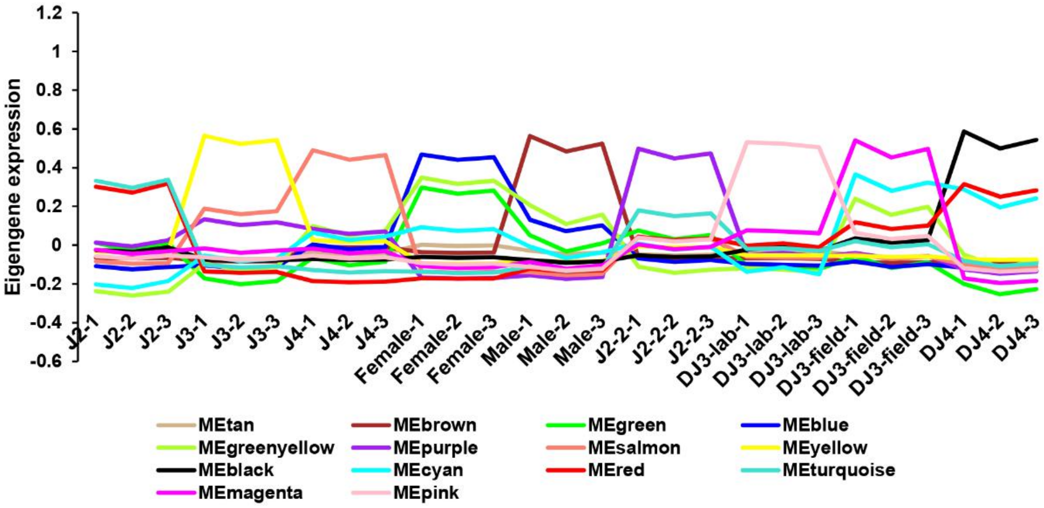

Figure S4 Module eigengene expression profile.

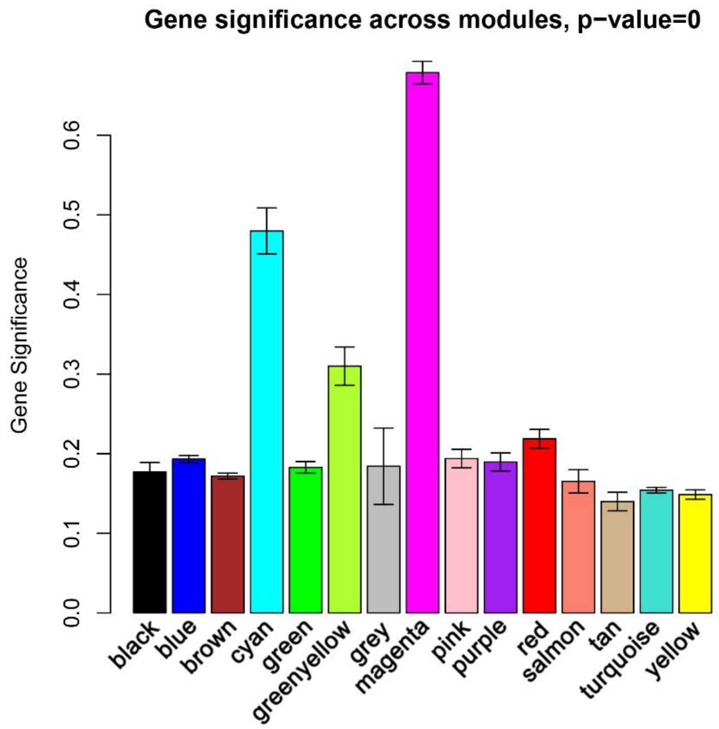

Figure S5. Bar plot of module significance defined as the mean gene significance across all genes in the module to the DJ3-lab trait. The magenta module is the most promising.

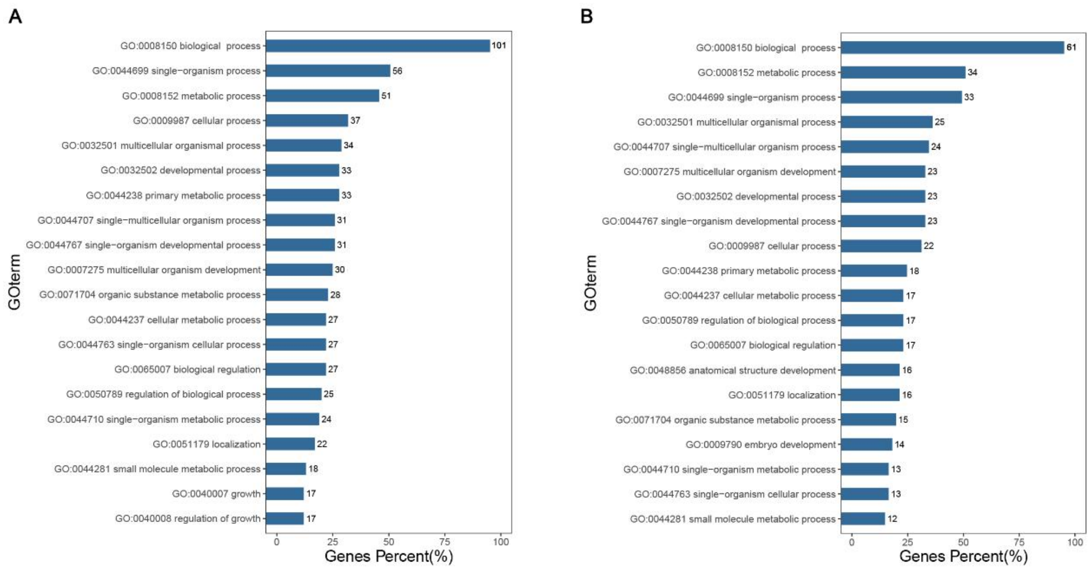

Figure S6. GO enrichment analysis (Biology Process) of the genes in the pink and magenta modules. **A:** Module pink. **B:** Module magenta.

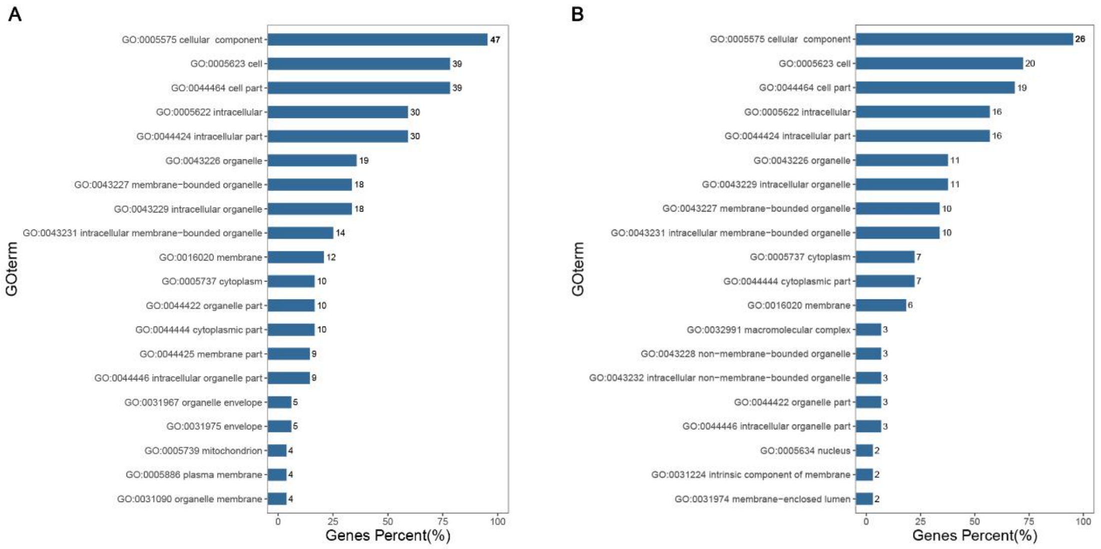

Figure S7. GO enrichment analysis (Cellular Component) of the genes in the pink and magenta modules. **A:** Module pink. **B:** Module magenta.

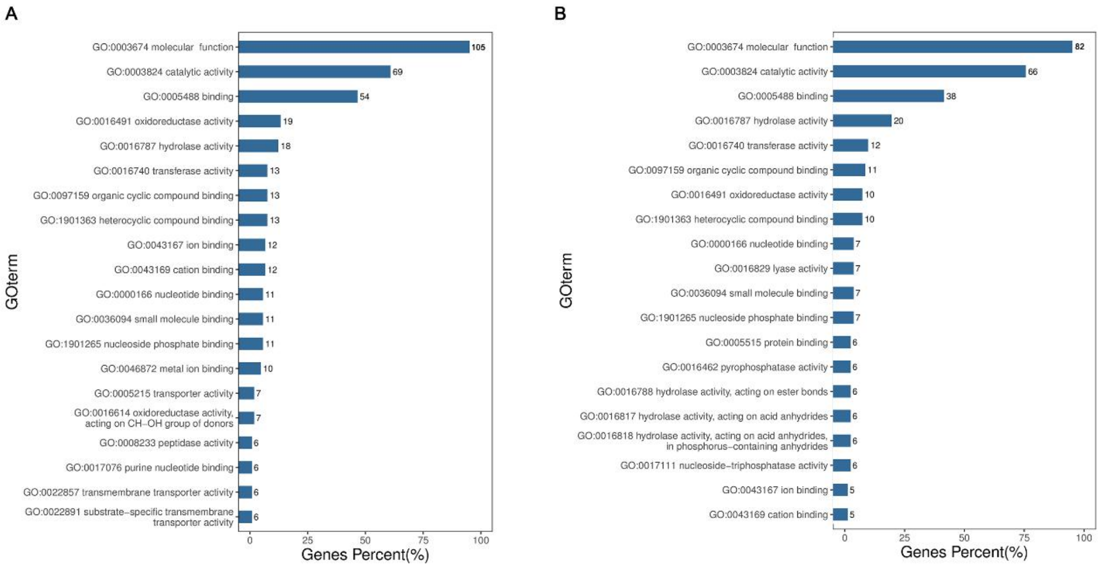

Figure S8. GO enrichment analysis (Molecular Function) of the genes in the pink and magenta modules. **A:** Module pink. **B:** Module magenta.

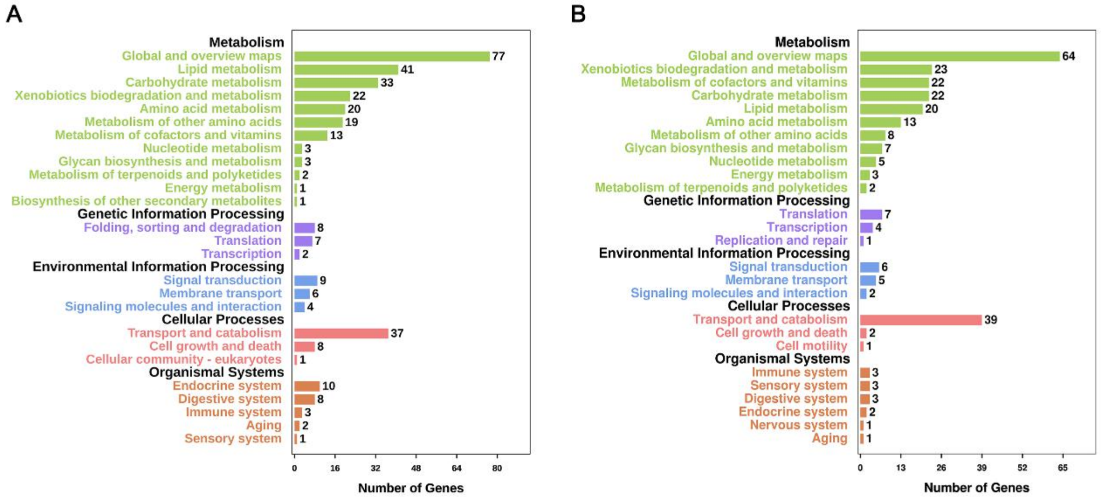

Figure S9. KEGG enrichment analysis of the genes in the pink and magenta modules. **A:** Module pink. **B:** Module magenta.

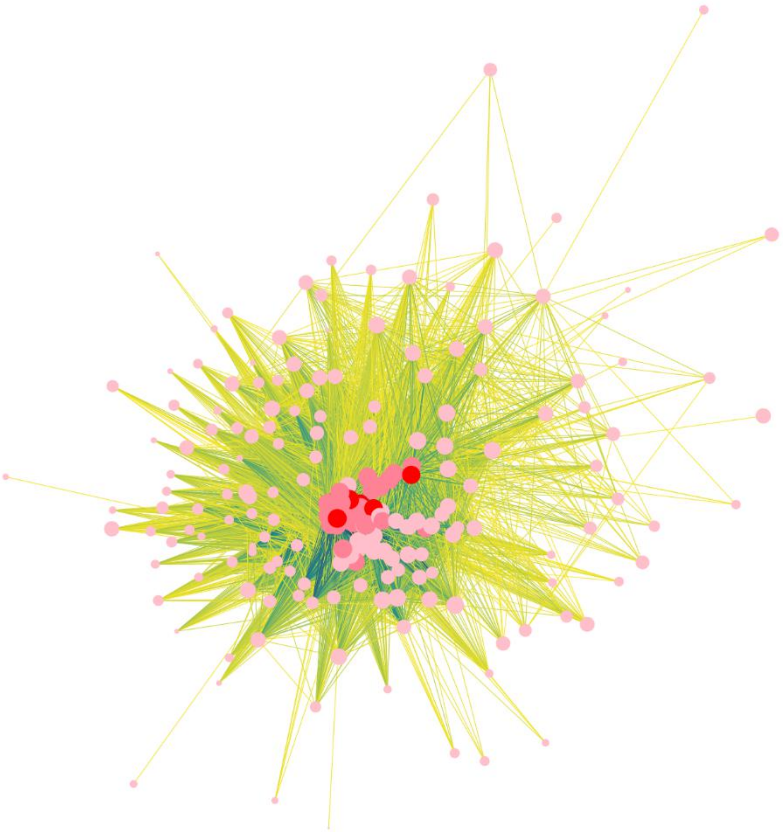

Figure S10. Visualization of pink module (weight value>0.3). Prefuse Force Directed Layout was applied based on weight value. Size of the dots represents IC (from17.3445 to 159.5326). Deep pink highlights the top 30 hub genes. Red highlights 6 hub genes in the pink module with extremely high homology to cysteine protease family cathepsin 1.
